# Scalable population-level modelling of biological cells incorporating mechanics and kinetics in continuous time

**DOI:** 10.1098/rsos.180379

**Published:** 2018-08-01

**Authors:** Stefan Engblom, Daniel B. Wilson, Ruth E. Baker

**Affiliations:** 1 Division of Scientific Computing, Department of Information Technology, Uppsala University, 751 05 Uppsala, Sweden; 2 Wolfson Centre for Mathematical Biology, Mathematical Institute, University of Oxford, Radcliffe Observatory Quarter, Oxford OX2 6GG, UK

**Keywords:** continuous-time Markov chain, computational cell biology, cell population modelling, notch signalling pathway, avascular tumour model

## Abstract

The processes taking place inside the living cell are now understood to the point where predictive computational models can be used to gain detailed understanding of important biological phenomena. A key challenge is to extrapolate this detailed knowledge of the individual cell to be able to explain at the population level how cells interact and respond with each other and their environment. In particular, the goal is to understand how organisms develop, maintain and repair functional tissues and organs. In this paper, we propose a novel computational framework for modelling populations of interacting cells. Our framework incorporates mechanistic, constitutive descriptions of biomechanical properties of the cell population, and uses a coarse-graining approach to derive individual rate laws that enable propagation of the population through time. Thanks to its multiscale nature, the resulting simulation algorithm is extremely scalable and highly efficient. As highlighted in our computational examples, the framework is also very flexible and may straightforwardly be coupled with continuous-time descriptions of biochemical signalling within, and between, individual cells.

## 1. Introduction

Development, disease and repair all require the tightly coordinated action of populations of cells. In almost every case a plethora of interacting components, acting on a range of spatial and temporal scales, combines to drive the observed tissue-level behaviours. Research in the biosciences is now advanced to the stage where we have sufficient understanding of intercellular processes to build relatively sophisticated models of a wide range of cellular behaviours. A promising approach to generate, test and refine hypotheses as to the relative contributions of various mechanisms to tissue-level behaviours is that of cell-based computational modelling: individual cells are explicitly represented, each cell has a position that updates over time, and cells may also have an internal state or programme determining their behaviour. Cell-based models hold great potential in this regard because they can naturally capture both stochastic effects and cell–cell heterogeneity, and they can be used to explore tissue-level behaviours when complex hypotheses on the cellular scale prevent straightforward continuum approximations at the tissue level. Recent applications of cell-based models to study population-level behaviours include embryonic development [1–5], wound healing [6–8] and tumour growth [9–13].

Multiple cell-based modelling approaches exist, and they can be categorized according to their approaches to representing cell positions as being either on- or off-lattice. In the on-lattice approach, space is divided up into a discrete grid of lattice sites. A common type of on-lattice model is the cellular automaton, in which each cell occupies a single lattice site and attempts to move to a new site at each time step according to a set of update rules. This volume exclusion rule can be relaxed to allow multiple cells per lattice site, depending on the level of spatial description required by the problem under consideration. Position update rules typically take into account the number and type of neighbouring cells, and can also depend on other information associated with lattice sites, such as nutrient or signalling factor concentrations [14]. Hybrid models often use systems of ordinary differential equations (ODEs) or partial differential equation (PDEs) to model the evolution of biochemical concentrations (e.g. [10,11,15,16]). Position update rules can also be stochastic, so that cells move according to, e.g. a biased random walk [17–22]. In most cases, a regular lattice is used, for example, square or hexagonal, but unstructured lattices have also been used in several cases (see [23,24] and references therein). A different on-lattice technique is the cellular Potts model, wherein each cell is allowed to occupy multiple lattice sites, and energy minimization is used to propagate the shape of each cell over time. The cellular Potts model has been used to study biological processes ranging from cell sorting [25,26] and morphogenesis [27,28] to tumour growth [29,30], and methods to provide a macroscopic limit to these models have been provided [31,32].

A basic off-lattice approach to cell-based modelling is the cell-centre-based model which assumes cells are, in effect, point particles that interact with each other via some specified potential function [11,33,34]. Meanwhile, in vertex models [35–37] cell populations are modelled as a tessellation of polygons or polyhedra, whose vertices move owing to forces originating from the cells, while in immersed boundary models [12,38–40] cell boundaries are represented as a set of points that move like elastic membranes immersed in a fluid. Other cell-based models take the subcellular composition of cells into account, such as the subcellular element model [41] or the finite-element vertex model [42].

Each cell-based modelling approach has specific advantages and disadvantages, and incorporate different representations of both biochemical signalling and biomechanics [16,39,43–46]. For example, an immersed boundary model allows a detailed representation of cell shapes, but this benefit comes at an increased computational cost in comparison to other methods. Cellular Potts models are very versatile in the possible effects that can be modelled but, because updates are controlled via a Metropolis-type algorithm together with a user-specified Hamiltonian, time can only be measured in terms of Monte Carlo steps. When modelling a specific application, it is necessary to weigh the benefits of the existing cell-based models against each other in the context of the specific application. A comprehensive review of mechanocellular models, including a table that compares the different modelling approaches, is provided in [43,45,47] and a comparison of different model output is detailed in [45,46].

In this work, we will focus on lattice-based approaches that allow a user-specified maximum number of cells to occupy each lattice site [14,23,48]. On a given structured or unstructured tessellation of space, we develop constitutive equations governing the dynamics of the cell population. As in the cellular Potts model, our update rules are stochastic and are established from global calculations. An important difference, however, is that our simulations take place in continuous time, thus allowing for a meaningful coupling to other continuous-time models. Our rationale for developing this approach is a desire to be able to quickly and efficiently simulate three-dimensional tissues consisting of large numbers of cells, with inclusion of biomechanical effects, intercellular signalling and external inputs. Since our approach is to base the modelling of cell biomechanics on the Laplace operator over the spatial tessellation, we refer to our method as discrete Laplacian cell mechanics (DLCM). Relying on the discrete Laplace operator is advantageous because one may make use of highly developed and scalable numerical methods to evolve the biomechanical details of the cell population over time. An important advantage provided by our highly efficient framework is the potential ability to conduct parameter sensitivity analysis, parameter inference and model selection for cell-based models; these are increasingly important research tools in this era of quantitative, interdisciplinary biology.

The outline of this work is as follows: in §2, we detail the model and its computational implementation; in §3, we describe four examples that showcase the use of our modelling framework; and in §4, we summarize our results and discuss avenues for future exploration.

## 2. Methods

The method we propose is developed by distributing the cells onto a grid of *voxels* and defining a suitable physics over this discrete space. The Laplace operator emerges as a convenient and basic choice to describe evolution of the biomechanics of the population, but more involved alternatives could also be employed in its place. We enforce a bound on the number of cells per voxel such that processes at the scale of individual cells may be meaningfully described on a voxel-local basis. For the simulations performed in this paper the voxels contain a maximum of two cells, but larger carrying capacities than this can also be supported. The choice of discretization (and so the maximum number of cells that can be accommodated in any voxel) should be made on a case-by-case basis, taking into account the need to balance computational complexity with the extent to which data on individual-cell-level processes are available. By evolving the individual cells via discrete PDE operators, e.g. the discrete Laplacian, processes at the population level are connected in an efficient and scalable way to those taking place inside the individual cells. In §2.1, we offer an intuitive algorithmic description of our framework, and a more formal development is found in §2.2.

### 2.1. Informal overview of the modelling framework

**Figure 1.**
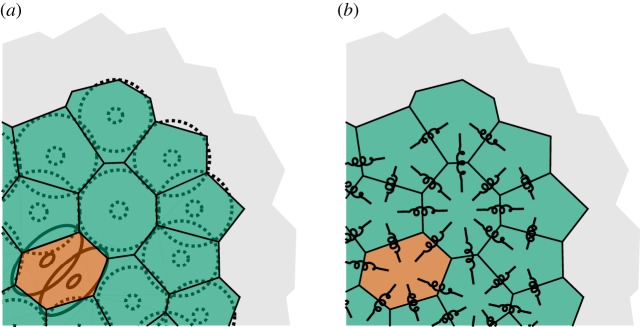
Schematic explanation of the numerical model. An unstructured Voronoi tessellation (*a*) with *green* voxels containing single cells and a *red* voxel containing two cells. The modelling physics for the cellular pressure can be thought of as if the pressure was spread evenly via linear springs connecting the voxel centres (*b*). The grid here is unstructured, but the same derivation is used for regular, e.g. Cartesian and hexagonal, grids.

We consider a computational grid consisting of voxels *v*_*i*_, *i* = 1, …, *N*_vox_, in two or three spatial dimensions. We make no assumptions as to whether the grid is structured or not, however, we do require that a consistent Laplace operator may be formed over the grid. Each voxel *v*_*i*_ shares an edge with a neighbour set *N*_*i*_ of other voxels. In two dimensions, each voxel in a Cartesian grid has four neighbours and on a regular hexagonal lattice, each voxel has six neighbours. On a general unstructured triangulation, each vertex of the grid has a varying number of neighbour vertices and, in this general and flexible case, the voxels themselves can be constructed as the polygonal compartments of the corresponding dual Voronoi diagram (figure 1).

At any given point in time, the voxels are either empty or may contain a certain number of cells. If the number of cells is at or below *the carrying capacity*, the system is assumed to be at steady-state. Hence in the absence of any other processes, the cell population is then completely static. If the number of cells in one or more voxels exceeds the carrying capacity, the cells push each other and exert a cellular pressure. Eventually, this non-equilibrium state is changed by an event, for example, one of the cells moves into a neighbouring voxel, and the pressure is redistributed. This goes on until, possibly, the system relaxes into a steady state.

What is the relevant constitutive equation for this cellular pressure? We make a more detailed investigation of this in §2.2, but chiefly for an isotropic medium and a scalar potential, thus essentially assuming the pressure to be spread evenly as in figure 1*b*, the answer is that the pressure is distributed according to the negative Laplacian, with source terms for all voxels where the carrying capacity is exceeded.

At any instant in time, a pressure gradient between two neighbouring voxels induces a force that, in turn, can cause the cells within the voxels to move. The rate of this movement is proportional to the pressure gradient, with a conversion factor that may depend on the nature of the associated movement. For example, it may be reasonable to assume that a cell may move into an empty voxel or into an already occupied voxel with different rates per unit of pressure gradient.

The simulation method is event-based and takes the form of an outer loop over successive events, see algorithm ??, lines ??–??. Because the dynamics is driven by a discrete numerical PDE operator, i.e. the Laplacian in our context, we call the simulation method DLCM. It should be noted that it might be beneficial in certain situations to use a different driving PDE operator: for a concrete example, see §3.2.

For any given state of the cell population, the cellular pressure is calculated and the rates of all possible events are determined (lines ??–??). Here the sampling procedure of Gillespie [49] may be used; the sum of all the rates decides the time for the next event, and a proportional sampling next determines the event that happens (lines ??–??). Until the time of this next event, any other processes local to each voxel may be simulated in an independent manner (line ??). Finally, as the event is processed, a new cell population state is obtained and the loop starts anew.


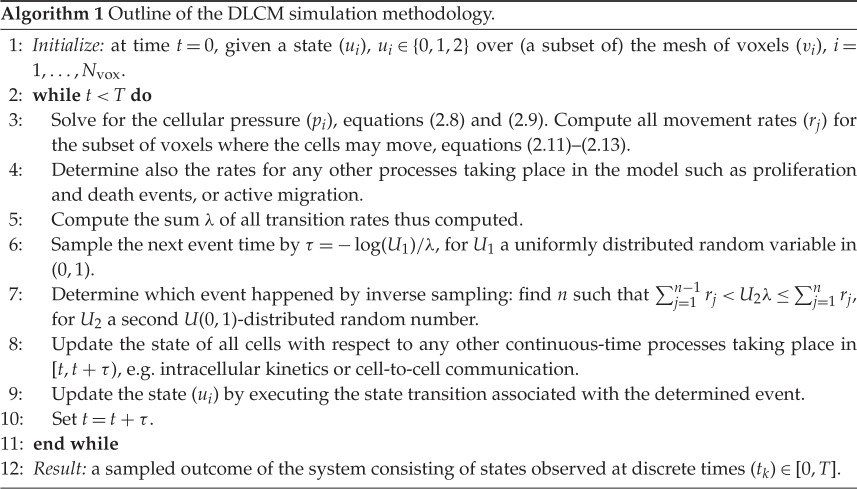


In summary, our new modelling framework is lattice-based, allows for a range of physical and biomechanical phenomena to be accurately described, and facilitates explicit modelling of cell size/excluded volume effects through the pressure-dependence of cell movements. The incorporation of stochasticity via the Gillespie algorithm renders it naturally able to represent the noise observed in most biological systems. Moreover, it is simple to simultaneously integrate either deterministic or stochastic models of biochemical reaction networks. Through the choice of sensible numerical linear algebra techniques, the framework is efficient and even complex models can be simulated in short times. This means that it will be possible to perform statistical inference of model parameters using quantitative experimental data together with techniques such as approximate Bayesian computation [50,51].

### 2.2. Formal description

We now develop the details of the DLCM framework. At some instant in time, let the grid (*v*_*i*_), *i* = 1, …, *N*_vox_, be populated with *N*_cells_ cells. In the interest of a transparent presentation, we only allow each voxel to be populated with *u*_*i*_∈{0, 1, 2} cells; other arrangements may also be useful, but a suitable physics for voxels populated *below* the carrying capacity should then depend on biological details such as the tendency of the cells to stay in close proximity to each other.

Owing to the spatial discretization and the discrete counting of cells, the task is to track changes over this chosen state space. In continuous time, this amounts to figuring out which cell will move to what voxel, and when it will move. This requires a governing physics defined over the discrete state. A continuous-time Markov chain respects the ‘memoryless’ Markov property and stands out as a promising approach, requiring only movement *rates* in order to be fully defined. Our model of the population of cells follows from three equations (2.1)–(2.3), understood and simplified under three assumptions, assumptions 2.1–2.3. We present each in turn as follows.

Let *u* = *u*(*t*, *x*) represent the cell density at time *t* and at the point *x*. We need to keep in mind that our numerical model is to be formulated on an existing grid of voxels (*v*_*i*_) containing a bounded (integer) number of cells *u*_*i*_. Hence the continuum limit *h* → 0 (of some suitable measure of the voxel size going to zero) is not meaningful. However, we shall use a continuous notation initially for ease of presentation. Our starting point is the continuity equation

2.1 $$\frac{\partial u}{\partial t}+\nabla \cdot I=0,$$

where *I* is the current, or flux. Since we are aiming at an event-based simulation we will later use equation (2.1) to derive rates for discrete events in a continuous-time Markov chain. To prescribe the current *I*, we now make a starting assumption.

**Assumption 2.1.**

The tissue is in mechanical equilibrium when all cells are placed in a voxel of their own.

Assumption 2.1 expresses the idea that small Brownian-type movements of each cell about its (voxel-) centre can be ignored. It does not exclude an additional description of any *active* movements, such as chemotaxis or haptotaxis. With sufficient conditions for equilibrium specified, it follows from assumption 2.1 that only doubly occupied voxels will give rise to a rate to move, and we will describe this increased rate as a pressure source. In the absence of any other units, we can set this pressure source to unity identically.

Let *p* = *p*(*t*, *x*) denote the cellular pressure at time *t* and position *x*, again using a continuous notation for variables which will be implemented on a discrete grid. Interpreting the current *I* as the result of a pressure gradient, we take the simple phenomenological model

2.2 I=−D∇p,

which, with the interpretation *D*≡*κ*/*μ*, the quotient between the permeability *κ* and the viscosity *μ*, is a form of Darcy's Law in porous fluid flow. Notably, Darcy's Law can be obtained from first principles using homogenization arguments [52]. Our use of equation (2.2), however, will be different as cells preserve their geometrical integrity and do not flow freely like a fluid. Rather, the current in equation (2.2) will be understood as a rate of the discrete event that a cell changes voxel.

To complete the model, we require a constitutive equation relating *u* and *p*. With the cellular pressure driven by sources in the form of overcrowded voxels, we provide a constitutive model of pressure evolution using the heat equation

2.3 $$\varepsilon \frac{\partial p}{\partial t}=\Delta p+s(u),$$

with *s*(*u*) a source function that will be prescribed below. Specifically, equation (2.3) follows by assuming an isotropic medium and a scalar potential pressure. For each voxel populated at or below the carrying capacity, there is no net flux of the potential and the divergence theorem implies the Laplace operator. For an overpopulated voxel, there is instead a net outward flux, then captured via the divergence theorem as a source term.

Equation (2.3) is a time-dependent PDE and would be complicated to handle within the current context. To move forward, we therefore need to bring in an additional assumption.

**Assumption 2.2.**

The cellular pressure of the tissue relaxes rapidly to equilibrium in comparison with any other mechanical processes of the system.

In cases where the biochemical kinetics of the individual cells also affect their mechanical behaviour, for example, via signal transduction or proliferation, assumption 2.2 entails that these processes must occur on a slower time-scale than the propagation of the cellular pressure. Importantly, assumption 2.2 simplifies equation (2.3) into the non-singular *ε* → 0 limit, the Laplacian equilibrium

2.4 −Δp=s(u).

The most immediate boundary conditions from equation (2.3) are

2.5 $$p{|}_{\partial \Omega_{D}}=0\;\text{(free boundary)}$$

and

2.6 $${\left(\frac{\partial p}{\partial n})\right|}_{\partial \Omega_{N}}=0\;\text{(solid wall)}.$$

The domain *Ω* understood here generally consists of the bounded subset of $R^{2}$ or $R^{3}$ which is populated by the cells. Its boundary ∂*Ω* can be written as ∂*Ω* = ∂*Ω*_*D*_∪∂*Ω*_*N*_, the Dirichlet and the Neumann boundaries, respectively, which can be chosen according to the specific biological problem under consideration. It is also possible to interpolate between the two boundary conditions

2.7 $${\left[\alpha p+\left(\frac{\partial p}{\partial n}\right)]\right|}_{\partial \Omega_{R}}=0\;\text{(semi-free boundary)},$$

to model, for example, an increasingly impenetrable cellular matrix as *α* → 0 by a homogeneous Robin condition. To simplify the presentation here, we employ homogeneous Dirichlet conditions (∂*Ω* = ∂*Ω*_*D*_) throughout the computational examples in §3.

**Figure 2.**
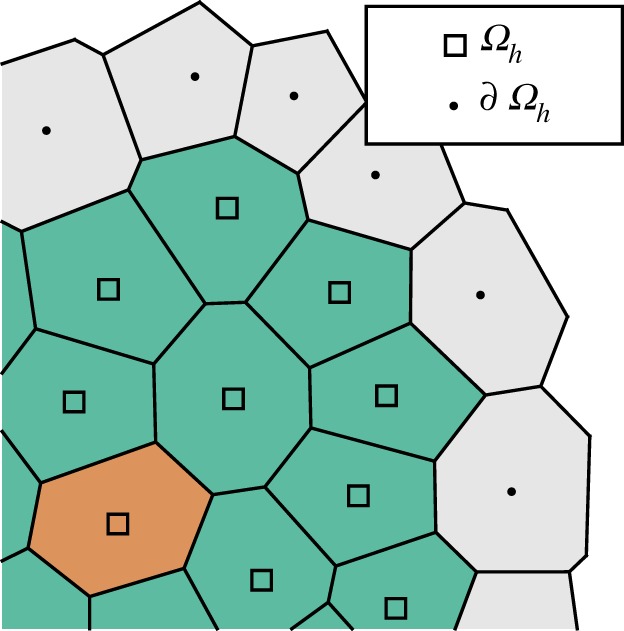
The definition of the discrete numerical domain *Ω*_*h*_, the set of populated voxels, and the numerical boundary ∂*Ω*_*h*_, the set of unpopulated voxels sharing an edge with *Ω*_*h*_.

We now re-interpret the developed model onto the given tessellation (*v*_*i*_), aiming specifically for a time-continuous and event-driven simulation. Denote by *Ω*_*h*_ the subset of voxels *v*_*i*_ for which *u*_*i*_&̸#x2009;= 0. Similarly, let ∂*Ω*_*h*_ denote the discrete boundary; this is the set of unpopulated voxels that are connected (i.e. share an edge) with a voxel in *Ω*_*h*_. See figure 2 for an illustration. We first consider the discrete version of equation (2.4):

2.8 $$-Lp=s(u),\;i\in \Omega_{h}$$

and

2.9 $$p_{i}=0,\;i\in \partial \Omega_{h},$$

where the source term is given by, in non-dimensionalized form, *s*(*u*_*i*_) = 0 for *u*_*i*_≤1 and *s*(*u*_*i*_) = 1 whenever *u*_*i*_ = 2, in view of the normalization *p* = 0 for the static case, following assumption 2.1. In equation (2.8), *L* is a discrete Laplacian over the currently active grid *Ω*_*h*_. The precise choice of (consistent) numerical method chosen to define *L* is not likely to have a strong influence on the model output since the Laplacian operator is quite forgiving to such details. In our experiments, we used a finite-element-based discrete Laplacian operator with linear basis functions and a lumped mass-matrix, which simplifies to traditional finite difference stencils on structured grids. It follows that the continuous interpretation of the source term is formally consistent with a Dirac delta function; in continuous language, *s*(*u*) = *s*(*u*(*x*)) is just $\sum_{u_{i}>c}\delta_{v_{i}}(x)$, where *v*_*i*_ here denotes the centre point of the *i*th voxel and *c* the carrying capacity.

We now go back to considering the induced movement of the cells according to the continuity equation (2.1). By equation (2.2), a pressure gradient gives rise to a proportional current; *I*∝ − ∇*p*. Let us write *I*(*i* → *j*) to denote the current from voxel *v*_*i*_ to neighbour voxel *v*_*j*_. In our discrete setting, this current is calculated by integrating the pressure gradient across the edge between the two voxels:

2.10 $$I(i\to j)\propto -\int_{v_{i}\cap v_{j}}\nabla p(x)\;dS=\frac{e_{ij}}{d_{ij}}(p_{i}-p_{j})=:I_{ij},$$

with *d*_*ij*_ the distance between voxel centres, *e*_*ij*_ the shared edge length, and where the simplification takes the discrete nature of the model into account by simply substituting a scaled difference for the gradient. In equation (2.10), the current is reversed (*j* → *i*) when the result is negative. It follows that, in the presence of a pressure source, a non-zero pressure gradient results everywhere except on solid (Neumann) boundaries. It remains to specify *D* in equation (2.2), including the subset of cells that are free to move as a result of this pressure gradient.

**Assumption 2.3.**

The cells in a voxel occupied with *n* cells may only move into a neighbouring voxel if it is occupied with less than *n* cells.

Let us write *R*(*e*) for the rate of the event *e* and use the notation *i* → *j* for the particular event that one cell moves from voxel *i* to *j*. Under our present framework, *u*_*i*_ is constrained to {0, 1, 2} and a total of three distinct cases emerges for the movement rates:

2.11 $$\begin{array}{rl} & R(i\to j;\;u_{i}\geq 1\text{, }u_{j}=0\text{, }v_{j}\text{ never visited})=D_{1}I_{ij}, \end{array}$$

2.12 $$\begin{array}{rl} & R(i\to j;\;u_{i}\geq 1\text{, }u_{j}=0\text{, }v_{j}\text{ visited previously})=D_{2}I_{ij} \end{array}$$

2.13 $$\begin{array}{rl} \;\mathrm{and}\; & R(i\to j;\;u_{i}>1\text{, }u_{j}=1)=D_{3}I_{ij}. \end{array}$$

Here, *D*_1_ is the conversion factor from a unit pressure gradient into a movement rate to a voxel *v*_*j*_ never visited before, *D*_2_ similarly, but into a voxel previously occupied, and *D*_3_ covers the ‘crowding’ case where a cell in a doubly occupied voxel enters a singly occupied voxel.

Although dependent on the specific application under consideration, we generally expect to have *D*_1_≪*D*_2_, as the extracellular matrix contained in previously unvisited voxels is usually thought to be less penetrable than that of previously occupied voxels. To understand the scale of *D*_3_, we note that from the divergence theorem

2.14 $$-\int_{\partial \Omega }(\nabla p\cdot n)\;dS=\int_{\Omega }s(u)\;dV.$$

**Figure 3.**
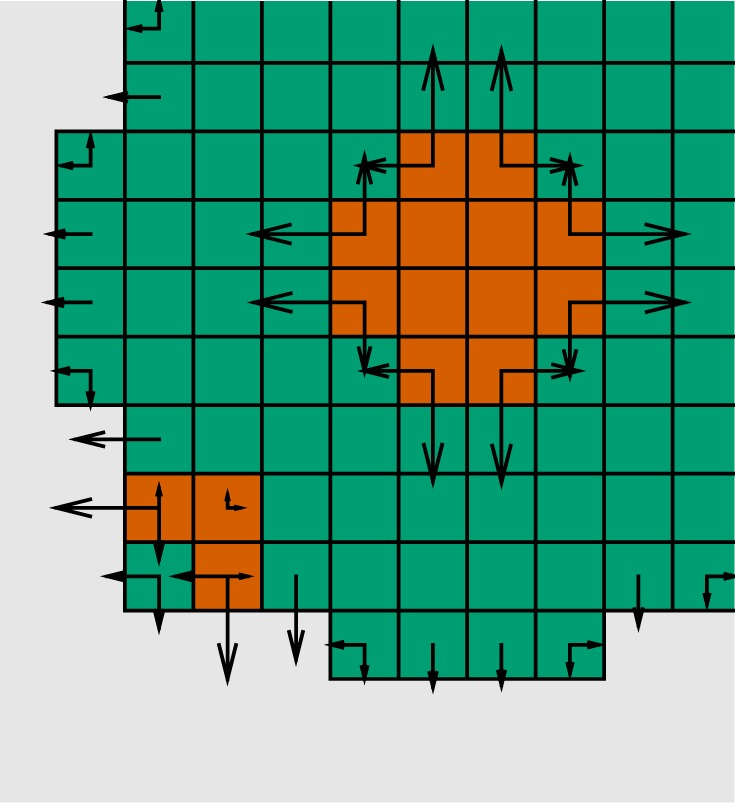
Schematic of the model: as in figure 1, *green* voxels contain single cells and *red* voxels contain two cells, giving rise to pressure sources. This pressure is propagated by a Laplacian operator over the voxels and induces a force, and hence a rate to move, for the cells in the subset of voxels indicated by the arrows. Cells in boundary voxels may move into empty voxels and cells in doubly populated voxels may move into voxels containing fewer cells. All arrows are drawn on a common scale.

In other words, the total pressure gradient over any closed surface ∂*Ω* is equal to the enclosed sources. With ∂*Ω* the boundary surrounding the whole-cell population, it follows that the ratio *D*_2_/*D*_3_ expresses the preference for events at the tissue boundaries (cells entering empty voxels) to events internal to the region (cells in doubly occupied voxels moving to an already occupied neighbouring voxel). For example, assuming the presence of a single internal source voxel and that *D*_2_/*D*_3_≡1, then the probability of a cell in the source voxel to move is the same as the total probability of a cell to move into any of the boundary voxels in ∂*Ω*. The rates in equations (2.11)–(2.13) are illustrated on a common scale with *D*_1_ = *D*_2_ = *D*_3_ in figure 3. Note that, on this Cartesian grid, by the integration over an edge in equation (2.10), the current is non-zero only along the cardinal directions up/down and right/left, respectively.

In addition to the rates in equations (2.11)–(2.13), one can easily extend the basic framework to include active cell movements and/or behaviours, as prescribed via additional rates and to be handled in lines ??–?? of algorithm ??. For example, a possible approach to include the effects of cell-cell adhesion is to define the resistance force for a cell moving from voxel *i* to *j* owing to cell–cell adhesion with cells in the non-empty voxel *k* as

2.15 $$a_{ijk}:=\alpha e_{ik}\min(0,r_{ij}\cdot r_{ik}),$$

for some adhesion constant *α* and for neighbour voxel pairs (*i*, *j*) and (*i*, *k*). Here *e*_*ik*_ is the edge length over which adhesion bonds are active and (***r***_*ij*_, ***r***_*ik*_) denote unit vectors pointing from voxel *i* to *j* and, respectively, from voxel *i* to *k*. The min-construct in equation (2.15) ensures that adhesion acts as a resistance (i.e. negative) force. Summing up the adhesion forces owing to all populated neighbour voxels of voxel *i* and scaling by the inter-voxel distance *d*_*ij*_ we thus evaluate the net adhesion current contribution as

2.16 $$\delta I_{\mathrm{adhesion}}(i\to j)=d_{ij}\sum_{k\in N_{i}}a_{ijk},$$

which is to be added to the current in equation (2.10). The resistance force works against the pressure-driven current in equation (2.10) to reduce cell movements.

Other options to capture similar effects could be to modify the pressure source function and/or the boundary conditions; the best choice is clearly dependent on the details of the modelling situation.

## 3. Results

We now present some computational results for the proposed modelling framework. In doing so, we focus on highlighting the three distinct advantages of our approach: (i) the mechanics is based on constitutive equations obeying a well-understood physics; (ii) the framework is fast and scalable, and therefore convenient to experiment with; and (iii), it is also flexible and allows for a seamless coupling to other processes of interest.

Throughout the examples below, we constructed our computational grid over a rectangular base geometry; we used structured Cartesian and hexagonal grids in two dimensions and in one case a Cartesian grid in three dimensions. The grid was initially populated by cells in the middle of the domain (cf. line ?? of algorithm ??). As previously mentioned, we relied upon a finite-element-based discrete Laplacian operator defined using linear basis functions and a lumped mass-matrix, implemented in the Matlab add-on package *PDE Toolbox* [53]. The discrete Laplacian was inverted using Matlab's built-in LU-decomposition (line ?? of algorithm ??); this was fast enough for our purposes and in all tests performed here. Larger models may well benefit from more advanced linear solver techniques. All code and the required data are available, and we refer the reader to ‘Data accessibility’ for details.

In §3.1, we look at the possible dependency of results on the underlying computational grid, and in §3.2, we investigate how to couple the cellular behaviour with signals in the local external environment. In §3.3, we couple the modelling framework with intracellular continuous-time processes, including cell-to-cell signalling and, finally, in §3.4 we demonstrate how the complex behaviours of a tumour model may be investigated.

### 3.1. Simulated cell population is free of grid artefacts

**Figure 4.**
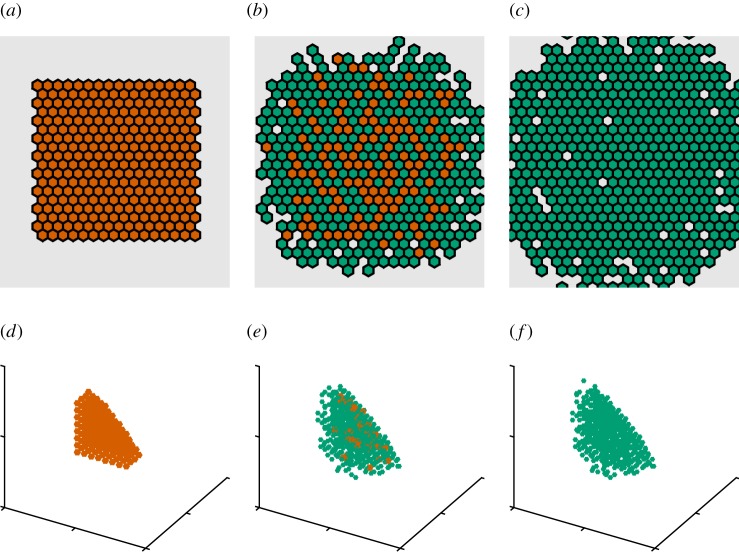
The set-up of the relaxation process experiment. (*a*) all voxels in a square initially contain two cells (red voxels), (*b,c*) (at 2.5 and 100 units of time, respectively): after relaxation the populated domain becomes approximately circular in shape and all voxels contain a single cell (green voxels). (*d*–*f*) The equivalent experiment in three space dimensions over a Cartesian grid (at 0.5 and 20 units of time, respectively). For visibility only, the voxels with coordinates (*x*,*y*,*z*) satisfying *x* + *y* + *z*≤0 are shown.

A disadvantage with grid-based methods and local update rules that has been highlighted in the literature is that grid artefacts may appear unless some care is exercised [54]. We test for grid artefacts by artificially forcing a number of cells into a square configuration and then relaxing the system to equilibrium (figure 4). In the absence of any other mechanical forces, clearly, the equilibrium state should be approximately circular.

**Figure 5.**
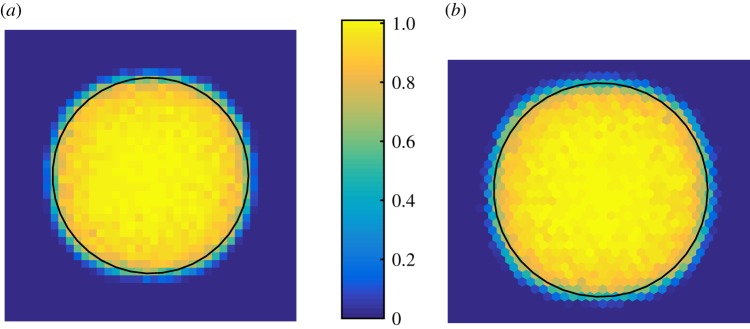
Average concentration (*N* = 100 trials) after relaxation from square initial data. (*a*) Cartesian grid and (*b*) hexagonal grid. The reference circle has the same area as the total cell population. Although a discrete effect is visible, there are no grid artefacts in the sense of preferred expansion directions.

In figure 5, we quantify the average cellular density across *N* = 100 independent runs of the model and find that, although a very small memory effect from the initial data are still visible (left/right and up/down), there is no dependence on the underlying grid except for the natural distortions owing to discreteness. This comes from the fact that the physics is based on well-understood constitutive equations and that we employ a grid-consistent discretization of the Laplacian (equations (2.4) and (2.8)).

### 3.2. Population-level behaviour via sensatory control

A notable extension of this basic framework is to model a population of cells that respond to external stimuli in their local environment. An advantage of the DLCM framework is that we are not restricted to considering a Laplacian operator but can freely choose other PDE operators, depending on the mechanics of the problem under consideration. As a test case, we take the two-dimensional migratory model of neurons responding to an inhibiting chemical known as Slit [55]. The cellular pressure, *p*, of the neurons is now described by the chemotaxis equation

3.1 $$\varepsilon_{p}\frac{\partial p}{\partial t}=\nabla \cdot (\nabla p+p\chi_{1}\nabla S)+s(u),$$

where *χ*_1_ is the chemotactic sensitivity of the neurons to the chemical *S*, i.e. the concentration of Slit. The parameter *ε*_*p*_ is taken to be small and represents our assumption that the pressure equilibrates on a faster time-scale than the movement of the neurons. The source term, *s*(*u*), is as defined previously. Equation (3.1) represents the movement of cells down the gradients of the Slit concentration. We set the domain for the experiment to be $x=(x_{1},x_{2})\in R^{2}$ and the equation governing the concentration of Slit is given by

3.2 $$\varepsilon_{S}\frac{\partial S}{\partial t}=D_{S}\Delta S-kS+Q\delta (x_{1}-X_{s}),$$

where *D*_S_ is the constant diffusivity of Slit, *k* is the rate of degradation and *Q* is the strength of the Slit source on the line *x*_1_ = *X*_*s*_. The parameter *ε*_S_ is again taken to be small and represents the assumption that the diffusion of Slit occurs on a faster time-scale than the movement of the cells. Imposing assumption 2.2 thus allows us to solve the steady-state problem

3.3 $$-\Delta p=\nabla (p\chi_{1}\nabla S)+s(u)$$

and

3.4 $$0=D_{S}\Delta S-kS+Q\delta (x_{1}-X_{s}),$$

where the boundary conditions for the Slit chemical are

3.5 $$S(x_{1},x_{2})\to 0,\;\mathrm{as}|x_{1}|\to \infty$$

and

3.6 $$\frac{\partial S}{\partial x_{1}}\to 0,\;\mathrm{as}|x_{1}|\to \infty .$$

We note that equations (3.4)–(3.6) can be solved analytically to give

3.7 $$S(x_{1},x_{2})=\frac{Q}{2\sqrt{kD_{S}}}\;e^{-\sqrt{k/D_{S}}|x_{1}-X_{s}|}.$$

**Figure 6.**
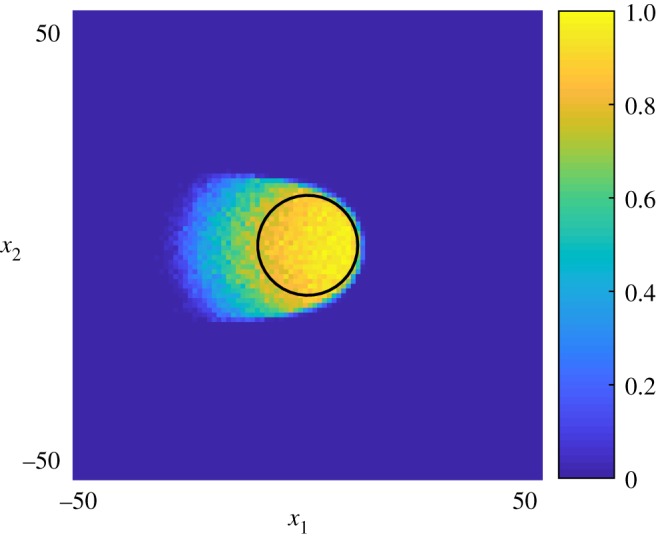
Average cell density (*N* = 100 trials) after relaxation from an initially circular explant (indicated by the black circle) with two cells in each voxel. The source of Slit is on the right-hand edge of the domain, at *x*_1_ = *X*_*s*_ = 50, and we see that, on average, cells move preferentially to the left, away from the source of Slit.

We also include a pressure-independent movement mechanism, representing the active motion of the cells owing to the chemo-repellent (see assumption 2.1). To include the chemotaxis-driven movement of a cell from voxel *i* to voxel *j* we derive the following current:

3.8 $$I(i\to j)=-\chi_{2}\int_{v_{i}\cap v_{j}}\nabla S(x)\;dS=\chi_{2}\frac{e_{ij}}{d_{ij}}(S_{i}-S_{j}),$$

where *χ*_2_ is a measure of the affinity of the cells to Slit for this type of pressure-independent movement. We initialize a circular region, with radius *r* = 10, of doubly occupied sites in the centre of the domain, where the voxel size is taken to be *h* = 1. We specify the parameters [*χ*_1_, *χ*_2_, *D*_S_, *k*, *Q*, *X*_*s*_] = [100, 5, 50, 0.1, 2, 50], and run 100 simulations until terminal time *T* = 1000. We present the average cell density profile in figure 6. As expected, on average the cell population moves away from the source of Slit, consistent with it being a chemo-repellent.

### 3.3. Continuous-time mechanics allows for seamless coupling to cellular signalling processes

A great flexibility with the proposed framework comes from the fact that additional processes may be simulated concurrently with the overall mechanics of the cell population (see algorithm ??, line ??). Notably, such processes may include cell-to-cell communication driving cell fate differentiation. To illustrate this, we consider the classical Delta–Notch intracellular signalling model [56], which produces pattern formation via a simple lateral inhibition feedback loop. With (*n*_*i*_, *d*_*i*_), respectively, the Notch and Delta concentrations within cell *i*, this model takes the dimensionless form

3.9 $$\begin{array}{rl} n_{i}^{\prime } & =f({\bar{d}}_{i})-n_{i}\\ \;\mathrm{and}\;d_{i}^{\prime } & =v(g(n_{i})-d_{i}), \end{array}\}$$

where ′ denotes differentiation with respect to time and

3.10 $${\bar{d}}_{i}=\frac{1}{|N_{i}|}\sum_{j\in N_{i}}d_{j},\;f(x)\equiv \frac{x^{k}}{a+x^{k}}\;\mathrm{and}\;g(x)\equiv \frac{1}{1+bx^{h}}.$$

We take parameters [*a*, *b*, *v*, *k*, *h*] = [0.01, 100, 1, 2, 2] and the average in equation (3.10) is taken over the set of neighbours *N*_*i*_ of the cell *i*. Additional stochastic noise terms may also be added to equation (3.9), but for simplicity we let the model be fully deterministic.

To produce a dynamic population, we let the cells in the middle of the region proliferate at a constant rate and we terminate the simulation when *N*_cells_ = 1000. The average in equation (3.10) is appropriately modified to account for any doubly occupied voxels. To get a more realistic contact pattern, a hexagonal grid was used; hence each voxel has six neighbours.

**Figure 7.**
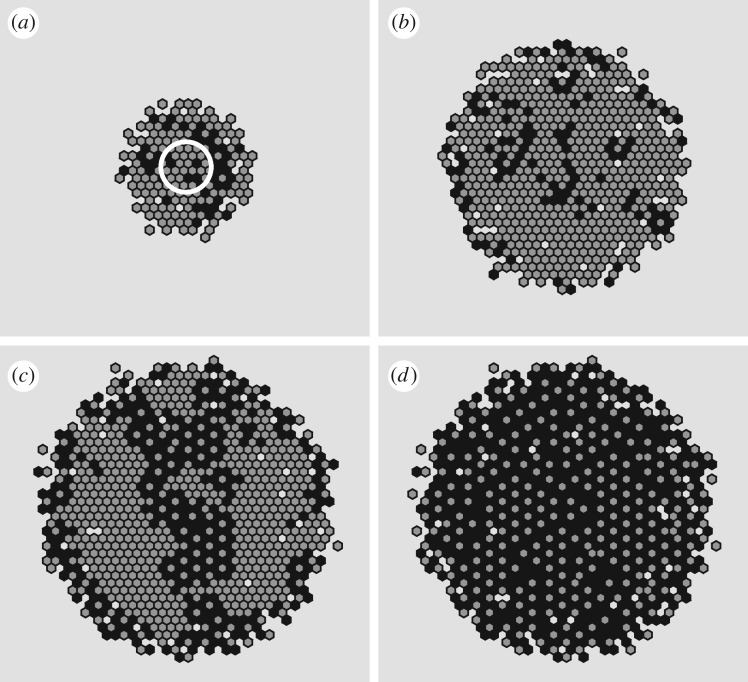
Coupling cellular signalling in continuous time: the dynamics of Delta–Notch when cells proliferate. *Black* cells indicate high Notch (*n*_*i*_ > 0.5) and *grey* cells indicate low Notch (*n*_*i*_≤0.5). (*a*) Initially cells are placed in a circular region (demarcated by the white circle) and all cells in this region are allowed to proliferate at a constant rate. (*b*) The population thus grows and a Delta–Notch signalling model is simulated concurrently. (*c,d*) The Delta–Notch model equilibrates after the cellular growth process has stopped. The times for these snapshots are *t* = [5, 40, 70, 100] units of time at unit proliferation rate, and the model was simulated using the Delta–Notch parameters described in the text.

**Figure 8.**
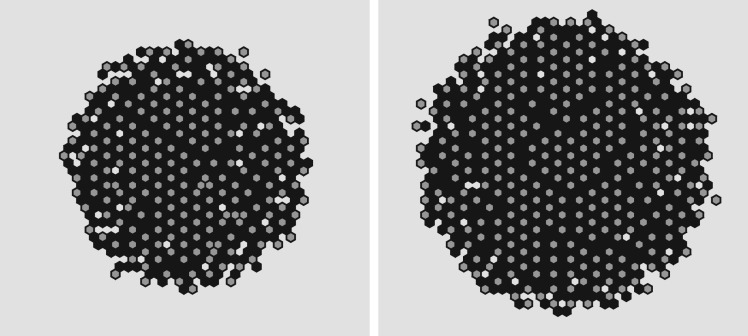
Delta–Notch dynamics simulated anew, but on a faster time-scale. Here, the process is essentially in continuous equilibrium as the tissue grows. These two frames correspond to (*b*) and (*c*) frames of figure 7.

In figure 7, a typical time-series of this model is summarized. Here the time-scale of the Delta–Notch dynamics, equation (3.9), is on a par with that of the proliferation process, so that their dynamics equilibrates after the tissue stops growing. A second simulation is summarized in figure 8, where the right-hand side of equation (3.9) has been scaled by a factor of 50 so that the Delta–Notch model is in quasi-steady-state as the tissue grows. The significant differences between the patterns could potentially be used, together with experimental data, to better understand how the time-scales for patterning compare with those of tissue growth.

### 3.4. Non-trivial dynamics emerges from the combination of grid-based physics and local behaviour

Finally, we use our proposed framework to build a model for avascular tumour growth. It is known that an important factor in the growth of tumours is the availability of oxygen to the tumour. In the case of an avascular tumour, there is no direct supply of oxygen to the tumour itself, but instead oxygen in the surrounding tissue diffuses into the tumour. We consider the tumour to be a growing population of cells that can potentially proliferate and/or die over the course of the simulation. We extend the occupancy of voxels such that *u*_*i*_∈{ − 1, 0, 1, 2}, where *u*_*i*_ = − 1 corresponds to a dead cell occupying a voxel. The cells at the boundary of the tumour can consume oxygen and proliferate if the concentration of oxygen is sufficient. Further into the tumour, where the concentration of oxygen is generally lower, the cells continue to consume oxygen but can no longer proliferate; these are known as quiescent cells. Near the centre of the tumour the oxygen concentration is low enough that the cells die and eventually degrade; this region is known as the necrotic core.

As before we solve the discrete Laplace equation (2.8) for the pressure in each voxel, with homogeneous Dirichlet boundary conditions (2.9) on the boundary ∂*Ω*_*h*_, the collection of empty voxels that are adjacent to occupied voxels. We also have an equation for the oxygen concentration, *c*, which diffuses through the domain with sources at the fixed external boundary, ∂*Ω*_ext_, thus,

3.11 −Lc=−λa(u)

and

3.12 $$c_{i}=1,\;i\in \partial \Omega_{\mathrm{ext}},$$

where *λ* is the rate of consumption of oxygen for a single cell and *a*(*u*_*i*_) is the number of alive cells (*u*_*i*_∈{0, 1, 2}) in the *i*th voxel (doubly occupied voxels consume twice as much oxygen and empty voxels or those containing dead cells consume no oxygen). From these discretized equations, we can calculate all the different rates for the possible events. These events are as follows. A cell occupying its own voxel, *u*_*i*_ = 1, will proliferate at rate *ρ*_prol_ if *c*_*i*_ > *κ*_prol_, where *κ*_prol_ is the minimum oxygen concentration for proliferation to occur. A living cell will die at a rate *ρ*_death_ if *c*_*i*_ < *κ*_death_, where *κ*_death_ is the threshold oxygen concentration for cell survival. In the case of cell death, *u*_*i*_ = 1 is replaced with *u*_*i*_ = − 1. A dead cell, *u*_*i*_ =  − 1, can degrade at a constant rate *ρ*_deg_ to free up the voxel (*u*_*i*_ = 0) for other cells to move in to it. We also include the rates for cell movement as shown in equations (2.11)–(2.13).

**Figure 9.**
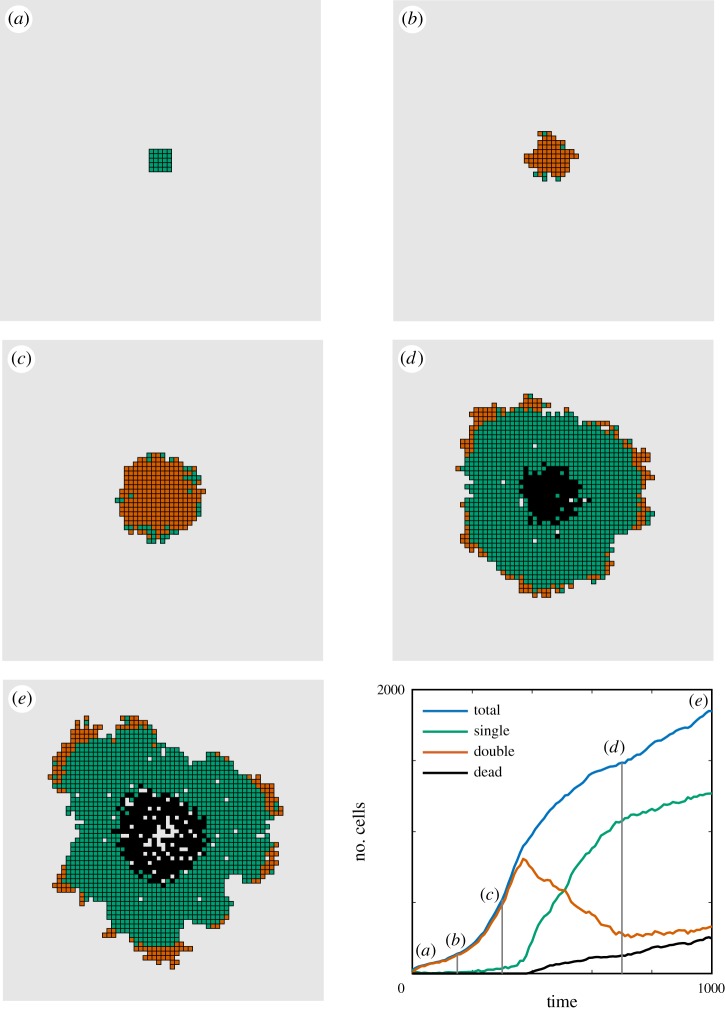
Snapshots of a realization of the avascular tumour model at times *t* = [0, 150, 300, 700, 1000]; the model parameters are as given in the text. (*a*) Initially cells are placed in a small 5 × 5 grid of singularly occupied voxels (green). (*b*) Cells have proliferated exponentially owing to an initial abundance of oxygen, and most voxels are doubly occupied (red). (*c*) The tumour grows at a quasi-linear rate, mostly voxels are still doubly occupied. (*d*) The lack of oxygen has forced a heterogeneity of cell types with a proliferating ring (red), an annulus of quiescent cells (green) and a necrotic core (black). The cell numbers appear to be plateauing. (*e*) Asymmetric protrusions form on the exterior of the tumour enabling the overall number of cells to continue to increase, thereby preventing a complete plateau.

In figure 9, we present snapshots from a realization of the model with parameters

$$\begin{array}{rl} & D_{1}=0.01\;s^{-1},\;D_{2}=25\;s^{-1},\;D_{3}=0.01\;s^{-1},\;\lambda =0.0015\;s^{-1},\\ & \kappa_{\mathrm{prol}}=0.65,\;\rho_{\mathrm{prol}}=0.125\;s^{-1},\;\kappa_{\mathrm{death}}=0.55,\;\rho_{\mathrm{death}}=0.125\;s^{-1},\;\rho_{\deg}=0.01\;s^{-1}, \end{array}$$

that demonstrates the evolution of the tumour into the classical regions of proliferating cells, quiescent cells and a necrotic core. We also present the evolution of the numbers of each cell type over the duration of the realization (figure 9, bottom right). The widely held view in the field is that a limited oxygen supply leads to a stable, finite-sized tumour [57–59]. Recent models for avascular tumour growth have been used to successfully predict the growth of tumour cell lines that exhibit sigmoidal growth curves [60]. Sigmoidal curves have three phases: for an initially small population of cells (figure 9*a*) in an abundance of oxygen we see exponential growth (figure 9*b*), which is then followed by a quasi-linear growth of proliferating cells (figure 9*c*). Eventually, the lack of oxygen causes three heterogeneous cell types to appear (figure 9*d*); a proliferating ring, a quiescent annulus and a necrotic core. It is here where the volume of the avascular tumour should begin to plateau, as observed in simulations (figure 9, bottom right (*d*)).

However, owing to a possible lack of surface adhesion in our model we observe a fourth phase emerge, where asymmetric protrusions appear on the surface (figure 9*e*) of the tumour which increases the total uptake of oxygen and hence a further incline in the total cell number (figure 9, bottom right). We sought a parametrization of our model that did not exhibit this fourth phase and instead produces a steady-state tumour, however we could not do so despite a systematic search of parameter space. We can navigate the parameter space as follows; firstly, select a user-specified finite-size tumour radius and with it solve the steady-state oxygen equation. Then the parameters (*λ*, *κ*_prol_, *κ*_death_) can be chosen to decide where the proliferating ring and necrotic core should lie. Next, initialize a population of cells at the required radius with some proliferation and death rates *ρ*_prol_ and *ρ*_death_, and temporarily let *ρ*_deg_ = ∞ such that dead cells instantly degrade. We can then alter the movement rates *D*_1_, *D*_2_ and *D*_3_ such that the number of cells proliferating is roughly equal to the number of cells being instantly absorbed in the necrotic core; this means that, on average, the size of the tumour will be constant. Finally, returning to the full avascular tumour model, we can vary the remaining parameters (*ρ*_prol_, *ρ*_death_, *ρ*_deg_) in an attempt to build a finite-size tumour. Despite this comprehensive exploration of the parameter space, which is only possible owing to the efficiency and versatility of the DLCM framework, at best we could only slow down the growth of the asymmetric protrusions by increasing *D*_2_ to be far greater than *D*_1_. That cells invade a new matrix at a slower rate appears to be a reasonable assumption here, but the exact separation in scales between *D*_1_ and *D*_2_ will need to be investigated on a case-by-case basis using cell trajectory data collected from relevant experiments. Thus, the current set of modelling assumptions, which only use passive physics, are not capable of developing the avascular tumour spheroids seen in experiments. Our results are in line with previous works [48,61] which both conclude that additional mechanisms are necessary in order for tumour growth to be stabilized. The suggested mechanisms involve the necrotic cells secreting chemicals that can either act as a growth inhibitor for healthy cells to become quiescent [61], or as a chemotactic signalling chemical to attract tumour cells to migrate back towards the necrotic core [48]. The inclusion of such mechanisms fits naturally into our proposed DLCM framework and will be implemented in future, more focused studies.

## 4. Discussion

The goal of this work was to provide an efficient, hybrid and multiscale computational framework for modelling populations of cells at the tissue scale. Our framework can harness either a regular or unstructured lattice, as required by the biological problem under consideration, and it draws upon a constitutive, mechanistic description of cellular biomechanics to drive expansion and/or retraction of the cell population as cells move and undergo proliferation and death. By assuming that the time-scale on which the tissue relaxes to mechanical equilibrium is much shorter than that of other mechanical processes, we are able to assume that the ‘cellular pressure’ within the population is governed by the Laplace equation with specified boundary conditions and source terms. We then use the pressure gradient within the tissue to derive rate equations for the movement of cells within the population. This enables us to propagate the model in continuous time, using an event-driven algorithm such as that originally proposed by Gillespie [49]. A significant advantage of our approach in this regard is that both discrete and continuous models of biochemical signalling can easily and flexibly be incorporated into the framework. This enables the user to develop hybrid and multiscale models that include constitutive descriptions of cellular biomechanics, inter- and intra-cellular signalling in an efficient and scalable framework. Our approach is flexible, computationally efficient and provides a consistent description of cellular mechanics. However, it does not, in the form presented here, allow for detailed tracking of individual cells, or a resolution of their shape, over time; other cell-based modelling approaches may be more suitable if such a spatial resolution is required [45,47].

We have demonstrated the use of our approach using four examples. First, we demonstrated that, as expected, a simple colony of proliferating cells relaxes isotropically. Second, we demonstrated how to integrate signals from the local microenvironment into cell movement laws, using the migration of cells within a neuronal explant exposed to a gradient in the secreted protein Slit as an example. Our third example showed that it is easy to integrate cell–cell signalling models into the framework, using the delta–notch lateral inhibition model as a test case. Finally, we developed a model for avascular tumour growth, wherein cell proliferation and death is controlled by the local oxygen concentration, and the cell population generates an oxygen profile within the tumour as the cells consume oxygen.

In summary, the real advance provided by our approach is the ability to quickly and efficiently simulate the behaviour of large populations of cells, in both two and three spatial dimensions. Our model is both hybrid and multiscale in nature; it can incorporate both continuum and discrete models of biochemical signalling both within and between cells. For the purpose of experimenting, we have developed a serial Matlab implementation of the model that employs a direct factorization of the discrete Laplace operator and, as such, scales conveniently to about 20 000 cells. Using a compiled language and parallelization, one could probably improve upon this figure to some extent. However, real savings in computing time will require optimal Laplace solvers that use, for example, algebraic or geometric multigrid techniques [62,63] that can take advantage of the incremental nature of the computational process when the domain evolves slowly. We leave this aspect of the implementation for future work.

In the modern era of biology, where quantitative data describing the evolution of cell populations and tissues can be collected with relative ease, we are now in a position to test and validate experimentally generated hypotheses using biologically realistic mechanistic models. However, these models need to be calibrated against the available data, and the sensitivity of model predictions to changes in model parameters needs to be explored. All of this requires repeated simulation of multiscale and hybrid cell-based models, the computational demands of which have to-date provided a barrier to significant progress. The DLCM method we outline here results in significant advances in our ability to efficiently simulate hybrid and multiscale cell-based models in two and three spatial dimensions, and, therefore, provides a very real opportunity to test and validate mechanistic models using quantitative data.

## Data accessibility

The computational results can be reproduced within release 1.4 of the URDME open-source simulation framework, available for download at www.urdme.org.
